# Characterization of Lipopeptide Biosurfactants Produced by *Bacillus licheniformis* MB01 from Marine Sediments

**DOI:** 10.3389/fmicb.2017.00871

**Published:** 2017-05-16

**Authors:** Yulin Chen, Shiliang A. Liu, Haijin Mou, Yunxiao Ma, Meng Li, Xiaoke Hu

**Affiliations:** ^1^Yantai Institute of Coastal Zone Research, Chinese Academy of SciencesYantai, China; ^2^School of Food Science and Engineering, Ocean University of ChinaQingdao, China; ^3^School of Veterinary Medicine, Louisiana State University, Baton RougeLA, USA

**Keywords:** *Bacillus licheniformis*, purification, bacteriostat, structure, LC-ESI-MS/MS, stabilization

## Abstract

Antibiotic resistance has become one of the world’s most severe problems because of the overuse of antibiotics. Antibiotic-resistant bacteria are more difficult to kill and more expensive to treat. Researchers have been studied on antibiotic alternatives such as antimicrobial peptides and lipopeptides. A functional bacteria MB01 producing lipopeptides which can be used as bacteriostat was isolated from the Bohai Sea sediments, which had been identified as *Bacillus licheniformis by* the morphological, physiological, and biochemical identification and 16s rDNA sequence. The lipopeptides produced by MB01 were determined to be cyclic surfactin homologs by LC-ESI-MS structural identification after crude extraction and LH-20 chromatography. [M+H]^+^ *m/z* 994, 1008, 1022, and 1036 were all the characteristic molecular weight of surfactin homologs. CID analysis revealed that the molecular structure of the lipopeptides was R_n_-Glu^1^-Leu/Ile^2^-Leu^3^-Val^4^-Asp^5^-Leu^6^-Leu/Ile^7^. The lipopeptides showed well resistance to UV light and the change of pH and temperature.

## Introduction

Pathogenic bacteria infections and contaminations and related diseases are the major concerns in public health throughout the world. Moreover, antibiotic resistance for pathogenic bacteria is an emerging crisis (Chan et al., 1989; Alvarez et al., 1998; Mead et al., 1999; Sutherland et al., 2004; Spellberg et al., 2008). A number of antibiotics alternatives have been proposed (Cheng et al., 2014). Antibacterial lipopeptides were originally found in the metabolic product of *Bacillus subtilis* (Perez et al., 1993; Ongena and Jacques, 2008) and they have good characteristics of the surfactants, which are surface active agents and can lower the surface tension. The lipopeptides are produced by members of bacillus as secondary metabolites and have both hydrophilic group (amino acid or peptides; di-or polysaccharides; anions or cations) and hydrophobic group (saturated or unsaturated fatty acid). Surfactins, iturins, and fengycins are the most important members of lipopeptides (Ongena and Jacques, 2008). More *Bacillus* species were found to be able to produce lipopeptides such as *Bacillus pumilus, Bacillus cereus, Bacillus thuringiensis*, and *Bacillus licheniformis* (Morikawa et al., 1992; Koumoutsi et al., 2004; Singh and Cameotra, 2004; Sun et al., 2006). The latest researches show that most of the bacillus can produce one type of lipopeptide and a few can produce two or three types of lipopeptides (Romero et al., 2007; Pecci et al., 2010).

Compared with traditional antibacterial substances, the lipopeptides cause less bacterial resistance, are more friendly to the environment since they produce no pollution, and have a wider range of bacteriostatic spectrum (Peypoux and Michel, 1992; Banat et al., 2000). The lipopeptides have been applied in many fields such as agriculture, medicine, food, and environmental protection, and gradually become a hot research field (Banat et al., 2000). However, few sources and the high cost of the lipopeptides limit their application. In this study a *Bacillus licheniformis* strain was isolated and the lipopeptides produced investigated by bacteriostatic test was proved to have significant antibacterial effects.

ESI/MS was applied to determine the molecular structures of all three main lipopeptides (Surfactins, iturins, and fengycins) (Pecci et al., 2010; Iatsenko et al., 2014). As one of the most important members of the lipopeptides family, the molecular structure of surfactins has the following characteristics: (1) a fatty acid chain linked with a seven-amino acids-peptide via its β-hydroxy; (2) the C-terminal amino acid residues of peptide that turns to cyclic compound; (3) positions 3 and 6 of N-terminal side of the peptide are Leu, position 5 is Asp, and position 2, 4, and 7 are non-determinate, generally Val, Leu or Ile (Horowitz and Griffin, 1991; Jacques, 2011). Collision-induced dissociation (CID) analysis has been applied to authenticate structures of lipopeptides homolog. By far, there are no reports of dipolymer or tripolymer lipopeptides produced by *Bacillus* (Li et al., 2008; Tosco et al., 2015; Bóka et al., 2016; Urajová et al., 2016).

The methods of separation and purification used in this study can provide a reference for a large scale and efficient preparation process of the lipopeptide compounds. The analysis results of the structures can also form the basis for further studies.

## Materials and Methods

### Materials

Twenty soil samples collected from Bohai Sea (119°38′E, 38°64′N) was stored at 4°C until analysis. *Escherichia coli, Vibrio cholerae, Vibrio parahaemolyticus, Vibrio harveyi, Pseudomonas aeruginosa, Staphylococcus aureus, and Proteus species* stored in the laboratory were applied as the target bacteria strains for potential antibacterial activity detection (Zhang and Austin, 2000; Zorrilla et al., 2003; Harth-Chu et al., 2009).

### Screening of Functional Bacteria

Ten grams of soil sample was transferred into a flask containing 100 mL sterile water to make the concentration of 10^-1^ sample suspension. The concentration of 10^-2^, 10^-3^, 10^-4^, 10^-5^, 10^-6^, 10^-7^, and 10^-8^ sample suspension were made by serial dilution. Sample suspensions at concentration of 10^-5^, 10^-6^, 10^-7^, and 10^-8^ were coated onto LB-Agar (Difco^TM^ LB Broth, Miller; Becton, Dickinson and Company; tryptone 10 g, yeast 5 g, NaCl 10 g, agar 15 g in 1 L distilled water, pH 7.2˜7.4) plates, and incubated at 37°C for 24 h (Morikawa et al., 1992; Savadogo et al., 2011; Zouari et al., 2016). Isolated colonies were numbered as B01, B02, B03, B04, etc. The isolated colonies with the agar blocks were taken with 5 mm diameter sterile punch after the colonies were backed up in another plate. *Vibrio parahaemolyticus* fermentation during the logarithmic phase was diluted to 10^8^ CFU/mL, then 100 mL of suspension was coated on the plate evenly to make *Vibrio parahaemolyticus* solid medium plate. These agar blocks were transferred onto *Vibrio parahaemolyticus* solid medium plates which had been punched in advance, and incubated at 37°C for 24 h (Urrea et al., 2011; Chen et al., 2016). Growth of colonies was continuously observed and the colonies with antibacterial activities were recorded.

Selected bacterial strains with antibacterial activities were inoculated into 500 mL LB fluid medium and incubated at 37°C in a rotary shaker (150 rpm) for 24 h. Supernatants were collected after centrifuge at 8000 ×*g* for 10 min and 6 mol/L HCl was used to adjust the pH to 2.0. Then the supernatants were sedimented for 6 h at ambient temperature. Sedimentation was collected after centrifuge at 8000 ×*g* for 10 min, then dissolved by methyl alcohol and the pH was adjusted to 7.0 (Lee et al., 2008; Bóka et al., 2016). The mixture was then stirred by magnetic stirrer at ambient temperature for 5 h. Sedimentation was removed after centrifuge at 8000 ×*g* for 20 min. Supernatant was dissolved in 50 mL sterile water for analysis after vacuum evaporation (Benitez et al., 2010; Pecci et al., 2010). Standardized concentration of the bacteria strain was adjusted to 10^8^ CFU/mL before applying on the plate. One hundred microliter of determinand was added into the ostiole of the plate which had been coated by detection target bacterial strain in advance and incubated at 37°C for 24 h (Hsieh et al., 2001). Diameter of antibiotic circle (DAC) of inhibition zones was measured, which served as a reference of the antibacterial activity.

### Taxonomic Identification of the Strain

The morphological, physiological, and biochemical characteristics were studied by using fresh culture following the regulation of *Bergey’s Manual of Determinative Bacteriology* (Senesi et al., 2001; Miranda et al., 2008).

### 16s rDNA Sequence Analysis

Bacterial genomes were extracted using an Ultra Clean Microbial DNA Isolation Kit (MoBio Laboratories, Carlsbad, CA, USA). Universal primers were used for amplification, 27F: 5′-GAGAGTTTGATCCTGGCTCAG-3′ and 1492R: 5′-GGTTACCTTGTTACGACTT-3′. The following PCR reaction protocol was applied: initial denaturation at 94°C for 4 min, 30 circles of 94°C for 40 s, 56°C for 50 s, 72°C for 100 s, and final extension at 72°C for 10 min. PCR products were then sent to Invitrogen Trading for sequencing. NCBI BLAST and multiple sequence alignment by Clustal X were analyzed and phylogenetic tree was constructed by using MEGA 5.0 (Tendulkar et al., 2007; Ki et al., 2009).

### The Preparation of Crude Bacteriostat and LH-20 Gel Chromatography Purification

Crude bacteriostat was extracted from MB01 fermented liquid using the above method and filtered for desalination by 3000 Da Millipore ultrafiltration tube, which was trapped in the upper part because of its micelles form (Ma et al., 2016). The sample was purified by LH-20 gel chromatography, using 60% methanol as the mobile phase and 1 mL/min flow rate (Nguyen et al., 2010; Gu et al., 2011; Li et al., 2012). The sample was collected at the peak of retention time 55.166 min, and then purified using rotary evaporation for the following antibacterial activity test.

### Separation and Identification of the Lipopeptide Compounds by LC-ESI-MS/MS

Purified substance was dissolved in methanol to reach the concentration of 1 mg/mL and passed through Agilent Zorbax SB C-18 rapid column to obtain mass spectrogram samples and surfactin standard substance (Kim P.I. et al., 2004). Gradient strategy method was as follows: 0–3.5 min, 60% A to 93% A; 3.5–20 min, keeping 93% A and 7% B (A, acetonitrile; B, ultrapure water); min pressure 0 bar, max pressure 400 bar, pressure stability 10 bar; injection volume 10 μL; syringe speed 8 μL/s; flush volume 800 μL. LC-MS full scan positive mode was performed in the range from *m/z* 200 to 2000. The peaks of standard substance and samples had some of correlation in retention time range of 7.0–7.5 min, 8.0–8.5 min, and 9–10 min. [M+H]^+^*m/z* 994, 1008, 1022, and 1036 were obtained by the alignment with standard substance.

### Minimum Inhibitory Concentration (MIC) Test

*Vibrio parahaemolyticus* were cultivated to logarithmic phase. Bacterial concentration was adjusted to 10^6^ CFU/mL. One hundred microliter of fermentation broth was added into each well of the 96-well plates. Different concentrations of 100 μL lipopeptide extracts were added by gradient dilution method to final concentrations 32, 16, 8, 4, 2, 1, 0.5, 0.25, 0.125, and 0.0625 μg/mL. Equal sterile water was used as blank control. Sterile culture media and indicator fermentation media (no lipopeptide) were used as negative control and positive control, respectively. The 96-well plates were incubated at 37°C for 24 h before examination.

### Antibacterial Spectrum Test

Quadruple MIC lipopeptides were applied, respectively, to operate antagonism of functional bacterial strain with *Escherichia coli, Vibrio cholerae, Vibrio parahaemolyticus, Vibrio harveyi, Pseudomonas aeruginosa, Staphylococcus aureus, and Proteus species* for bacteria antibacterial activity test. Vernier caliper was used to measure DAC.

### Antimicrobial Resistance Test of the Lipopeptides

In order to confirm energy loss and retention under various conditions, residual rate was defined as follows. Residual rate (%) = DAC (experimental group)/DAC (control group) × 100% (control group: quadruple MIC new lipopeptides; experimental group: quadruple MIC lipopeptides under the conditions of different treatments).

### Temperature Stability Test

Quadruple MIC lipopeptides were placed at 40, 50, 60, 70, 80°C, respectively, for 10 days, also at 4°C (cold storage) and 25°C (room temperature) for 300 days. Bacteriostatic tests were applied to test temperature stability, with fresh quadruple MIC as contrast.

### pH and UV Resistance Test

For the potential industrial production and application, exploration of the production, shelf-life and storage conditions is necessary. Whether UV sterilization packaging in the production line would have an impact on the product or not is a question. In addition, considering the acid purification procedure, determination of its activity in close to neutral condition is needed. Different concentrations of hydrochloric acid and sodium hydroxide were applied to adjust the quadruple MIC lipopeptides pH from 1 to 14. The pH was adjusted back to neutral after 24 h. The concentration to quadruple MIC was adjusted again after desalting by dialysis. Quadruple MIC lipopeptides were placed under UV (20 W, 30 cm) for 10 days for UV resistance test.

## Results and Discussion

### Morphological Identification and 16S rDNA Sequence Analysis on MB01

**FIGURE 1:**
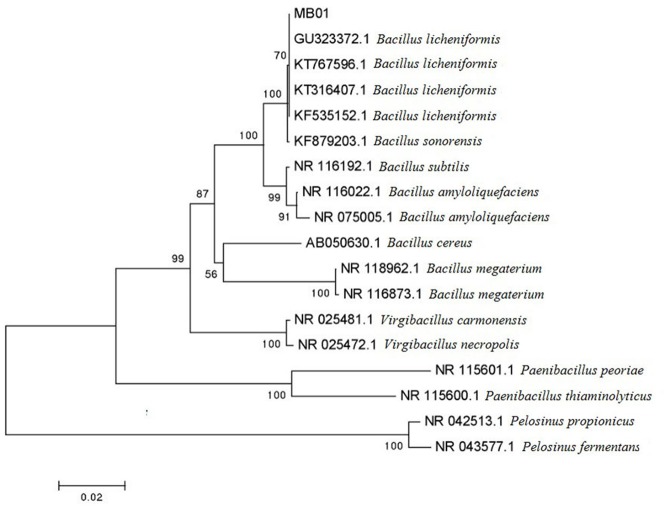
**Phylogenetic tree of MB01**.

MB01 is a gram positive rod shape bacterium and the colonies present circular, small red with rough surface. The result of physiological biochemical test is shown in **Table 1**. Voges–Proskauer reaction, nitrate reduction, indole and catalase test are positive. MB01 can utilize almost all of the seven different carbon substrates including D-xylose, D-aldohexose, D-glucose, L-arabinose, gelatin, citrate, and starch apart from tyrosine. The length of 16s rDNA is 1436 bp. MEGA (Molecular Evolutionary Genetics Analysis) was used to construct Neighbor-Joining phylogenetic tree in **Figure 1**. MB01 was 99% homologous identity to *Bacillus licheniformis* (GenBank: GU323372.1) with a reference of sequence alignment and phylogenetic tree. In consideration of the results of morphological characteristic and physiological biochemical test in **Table 1**, this strain was identified as *Bacillus licheniformis*, GenBank ID: KX499496, whose preservation number was CCTCC AB 2016262 in China Center for Type Culture Collection.

**Table 1:** The physiological and biochemical tests of MB01.

Test contents	Test results
Voges–Proskauer reaction	+
Nitrate reduction test	+
D-Xylose	+
D-Aldohexose	+
D-Glucose	+
L-Arabinose	+
Indole test	+
Citrate test	+
Gelatin hydrolysis	+
Amylohydrolysis	+
Tyrosine hydrolysis	-
Catalase test	+
Phenylalanine deaminase	-
Methyl red test	-
H_2_S	-

*+ indicates positive; - indicates negative.*

### LC-ESI-MS Analysis of the Lipopeptide Extracts

#### Mass Spectrogram Analysis

**FIGURE 2:**
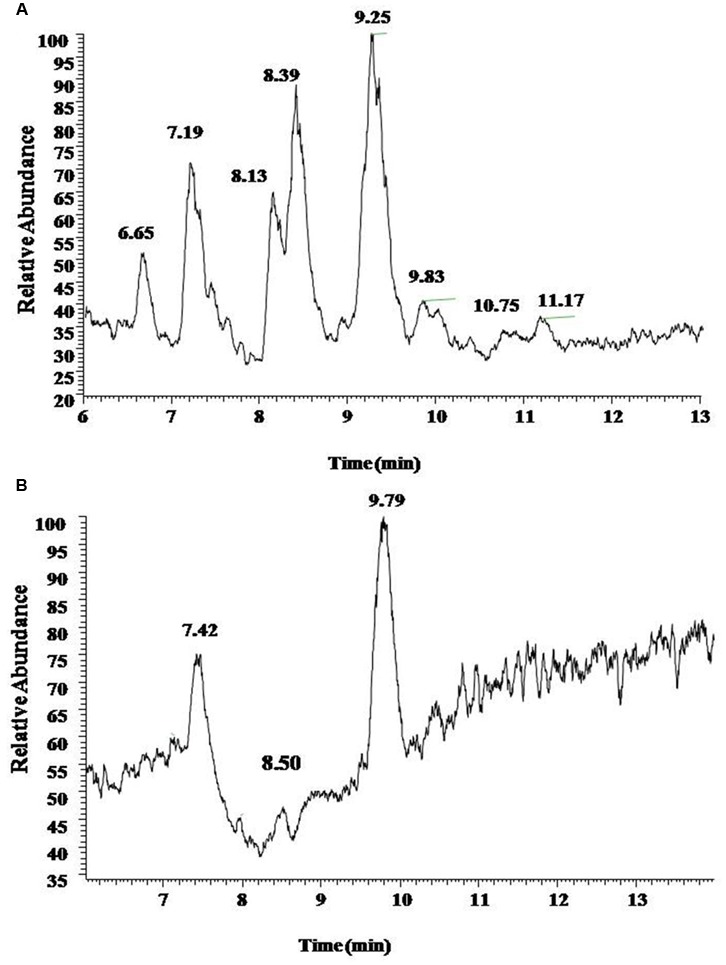
**The UV chromatogram of reference standards (A)** and sample **(B)**. Both reference standards and sample displayed Peak A at retention time 7.5 min, Peaks B and C at 8.5 and 9.5 min.

**FIGURE 3:**
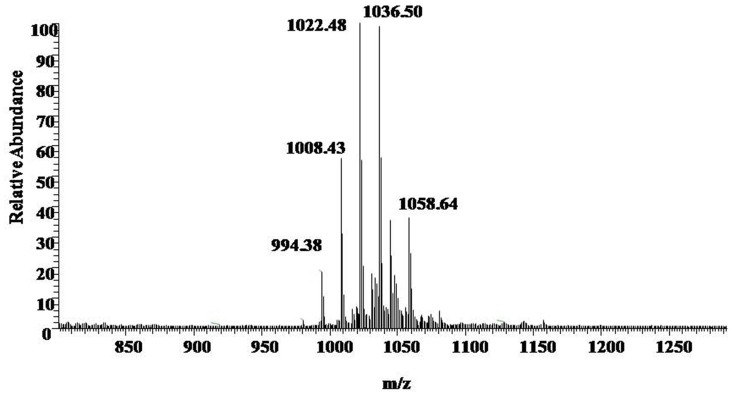
**ESI Full MS of Sample.** [M+H]^+^*m/z* 994, 1008, 1022, and 1036 are the characteristics of the molecular weight of surfactin.

**FIGURE 4:**
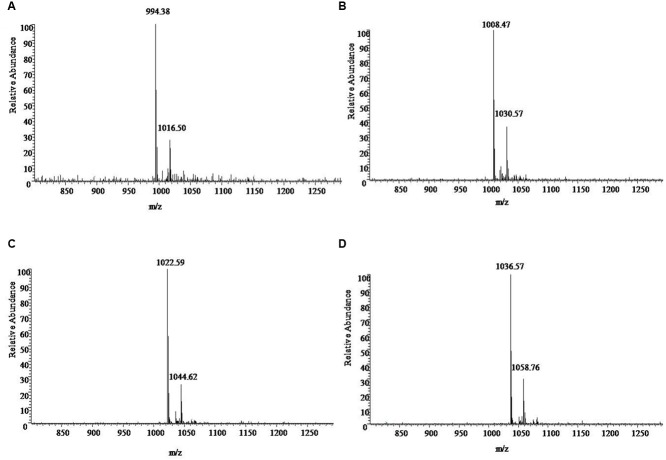
**The ESI-MS [M+H]^+^ chromatogram of sample. (A)** 994 is the characteristics of the molecular weight, with a surfactin precursor ion [M+Na]^+^ at *m/z* 1016. **(B)** 1008 is the characteristics of the molecular weight, with a surfactin precursor ion [M+Na]^+^ at *m/z* 1030. **(C)** 1022 is the characteristics of the molecular weight with a surfactin precursor ion [M+Na]^+^ at *m/z* 1044. **(D)** 1036 is the characteristics of the molecular weight, with a surfactin precursor ion [M+Na]^+^ at *m/z* 1058.

UV spectrograms revealed similar peaks in retention time of both surfactin standard substance and sample ranging at 7.0–7.5 min, 8.0–8.5 min, and 9–10 min in **Figure 2**, which could be speculated that the sample was likely to be surfactin compounds. Molecule-ion peaks appeared at [M+H]^+^ *m/z* 994, 1008, 1022, and 1036 in ESI Full MS of sample in **Figure 3** and UV spectrograms in **Figure 4**. Each molecule-ion peaks mass differed by 14 (equals to the mass of [CH_2_]), which illustrated that the four components might be homolog. Respectively, there were sodium peaks at [M+Na]^+^ *m/z* 1016, 1030, 1044, and 1058, beside [M+H]^+^*m/z* 994, 1008, 1022, and 1036. All the above peaks are the characteristic peaks of surfactin molecular weight.

#### MS/MS Spectrum Analysis

**FIGURE 5:**
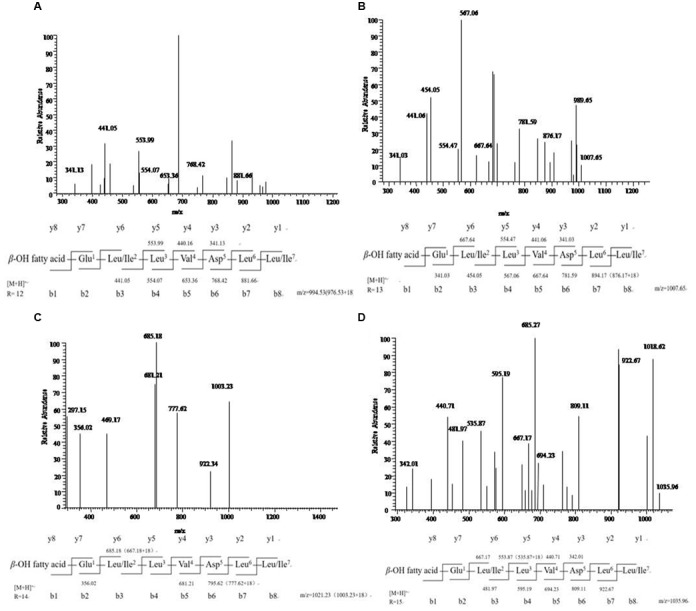
**LC-ESI-MS/MS spectrum of the cyclic surfactin precursors containing C_12_-C_15_ fatty acid chains. (A–D)** Cyclic surfactin precursors ion [M+H]^+^ at *m/z* 994, 1008, 1022, and 1036 containing a Glu^1^-Leu/Ile^2^-Leu^3^-Val^4^-Asp^5^-Leu^6^-Leu/Ile^7^ peptide.

The results of MS/MS spectrum of characteristic ions peaks, respectively, at [M+H]^+^ *m/z* 994, 1008, 1022, and 1036 were shown in **Figure 5**. From MS/MS spectrum of *m/z* 994 in **Figure 5A**, [M+H]^+^ *m/z* 976.53 was the dehydration ion peak [M-H_2_O]^+^ of *m/z* 994 and 112.34 (994 – 881.66 = 112.34) was exactly the fragment ion mass of Leu/Ile (b8). Starting from the b^+^ end, b^+^ ions fragments in order were 441.05 → 554.07 → 653.36 → 768.42 → 881.66. [M+H]^+^ *m/z* 441.05 was the total mass of ions fragments of R_12_, Glu, and Leu/Ile. The differences of above values were exactly the mass of ions fragments of Leu, Val, Asp, and Leu. Starting from the y^+^ end, [M+H]^+^ *m/z* 341.13 was the total mass of ions fragments of Asp, Leu, and Leu/Ile. The y^+^ ions fragments in order were 341.13 → 440.16 → 553.99. The differences of above values were exactly the mass of ions fragments of Val (y4) and Leu (y5). And 440.01 (994 – 553.99 = 440.01) was the total mass of ions fragments of R_12_, Glu, and Leu/Ile. From MS/MS spectrum of [M+H]^+^ *m/z* 1008 in **Figure 5B**, *m/z* 876.17 was the dehydration ion peak [M-H_2_O]^+^ of *m/z* 894.17. [M+H]^+^ *m/z* 1007.65 was the molecular ion peak. Also 113.48 (1007.65 – 894.17 = 113.48) was exactly the fragment ion mass of Leu/Ile (b8). Starting from the b^+^ end, [M+H]^+^ *m/z* 341.03 was the total mass of ions fragments of R_13_ and Glu. The b^+^ ions fragments in order were 341.03 → 454.05 → 567.06 → 667.64 → 781.59 → 894.17. The differences of above values were exactly the mass of ions fragments of Leu/Ile, Leu, Val, Asp, and Leu. Similar results could be drawn in **Figures 5C,D** to speculate the molecular structures of the lipopeptides homologs: R_n_-Glu^1^-Leu/Ile^2^-Leu^3^-Val^4^-Asp^5^-Leu^6^-Leu/Ile^7^ in **Table 2**. The different molecular weights depended on the different lengths of fatty acids chains. All of the four lipopeptides belonged to the surfactin family according to the LC-ESI-MS results.

**Table 2:** Assignment of the structures of lipopeptides by LC-ESI-MS/MS in this study.

No.	Mass (*m/z*)	Rt (min)	Family	Assignment	Sequence
1	994	6.53	Surfactin	C_12_[M+H]^+^	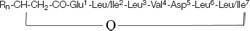
2	1008	7.40		C_13_[M+H]^+^	
3	1022	8.58		C_14_[M+H]^+^	
4	1036	9.79		C_15_[M+H]^+^	

#### Chemical Structural Formula of the Lipopeptides

**FIGURE 6:**
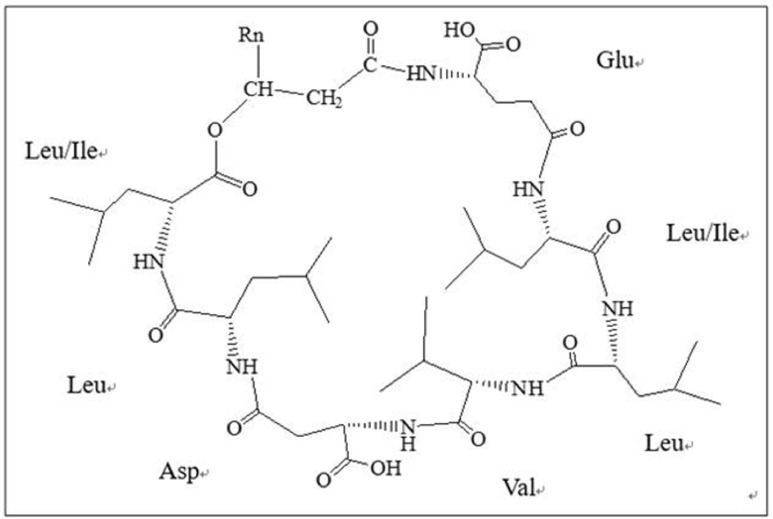
**Chemical structural formula of lipopeptides.** This lipopeptide consisted of a β-fatty acid chain and a peptide chain, with condensation of the carboxyl at the end of fatty acid chain and the hydroxyl of Leu/Ile at the end of peptide.

Chemical structural formula of the lipopeptides was shown in **Figure 6**. This lipopeptide consisted of a β-fatty acid chain and a peptide chain, with condensation of the carboxyl at the end of fatty acid chain and the hydroxyl of Leu/Ile at the end of peptide. There was a condensation of the carboxyl of Leu/Ile at the end of peptide and β-OH of fatty acid chain. This study revealed that the homologs of the lipopeptides had different length fatty acid chains including C_12_, C_13_, C_14_, and C_15_.

#### MIC Test Analysis

The MIC test revealed that minimal inhibitory concentration of the lipopeptides was 50 μg/mL. The growth of *Vibrio parahaemolyticus* had not been suppressed when the lipopeptides concentration was below 50 μg/mL.

#### Screening and Antibacterial Spectrum Test

MB01 that had been proved to have strong antibacterial activity was screened for bacteriostatic activity. MB01 had varying degrees of bacteriostasis to the gram positive and negative bacteria, through antibacterial spectrum tests in **Table 3**, which fully illustrated that this active substance produced by MB01 had broad-spectrum bacteriostat characters.

**Table 3:** The bacteriostatic activity of MB01 on indicator bacteria.

Indicator bacteria	*Escherichia coli*	*Vibrio cholerae*	*Vibrio parahaemolyticus*	*Vibrio harveyi*	*Pseudomonas aeruginosa*	*Staphylococcus aureus*	*Proteus species*
Bacteriostatic activity(DAC)	+++	+++	+++	+++	++	++	+

*+ DAC 1–2 cm, ++ DAC 2–3 cm, +++ DAC > 3 cm.*

#### Temperature Stability Test Analysis

**FIGURE 7:**
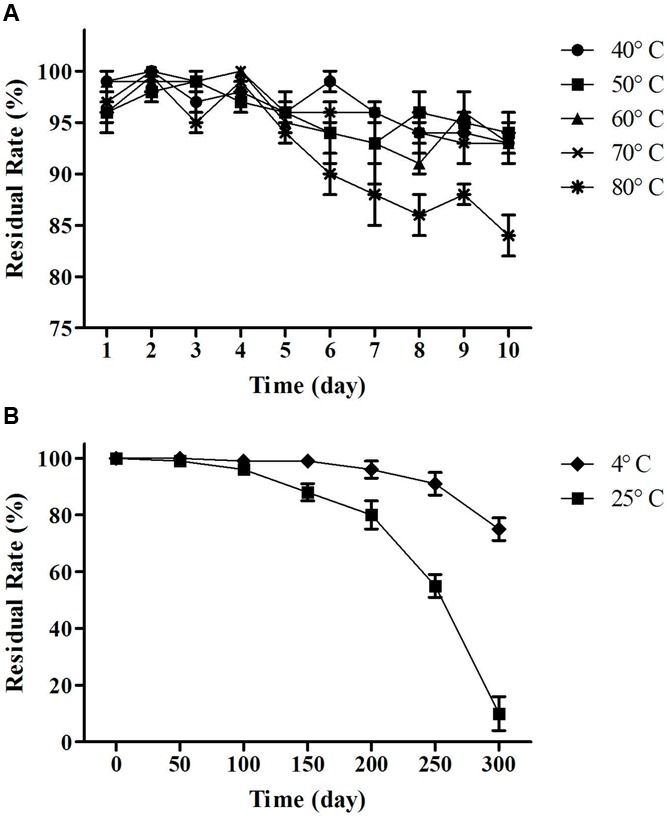
**Temperature stability test. (A)** Residual rate of change over time at 40, 50, 60, 70, and 80°C. **(B)** Residual rate of change over time at 4°C (cold storage) and 25°C (room temperature). Residual rate remained above 90% between 40 and 70°C and 85% at 80°C after 10 days. The lipopeptides had reserved more than 80% of activities after storage at 4°C for 300 days. The residual rate decreased to 59% after storage at 25°C for 250 days.

As shown in **Figure 7**, the activity of the lipopeptides from MB01 had almost no losses under 40–70°C for 10 days. Residual rate remained above 90% between 40 and 70°C after 10 days. However, residual rate dropped to 85% under 80°C after 10 days, which was significant lower the residual rate at 40–70°C (*p* < 0.05, **Figure 7**). The lipopeptides had reserved more than 80% of activities after storage at 4°C for 300 days. The residual rate decreased to 59% after storage at 25°C for 250 days. However, the lipopeptides lost almost all activity after 25°C storage for 300 days. The above results showed that the MB01 lipopeptides had very good storage stability at 4°C and room temperature.

#### Acid and Alkali and UV Resistance Test Analysis

**FIGURE 8:**
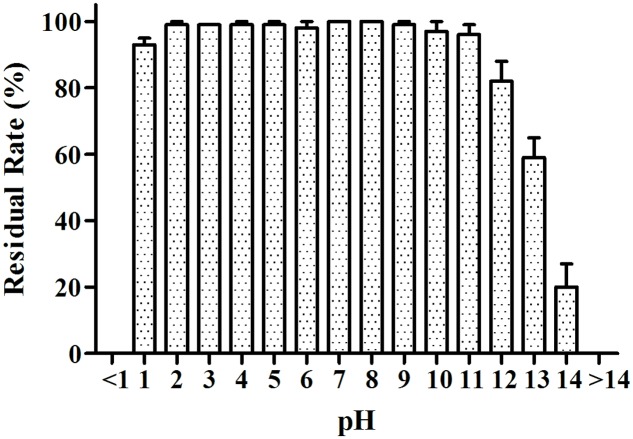
**pH resistance test. The activities of lipopeptides retained above 90% from pH 1 to pH 11**.

**FIGURE 9:**
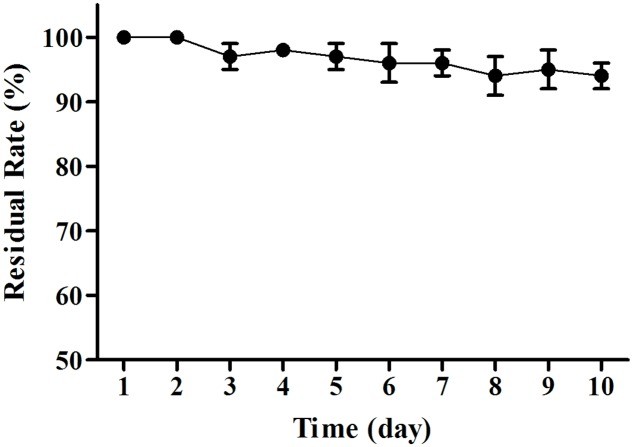
**UV resistance test**. The activities of lipopeptides retained above 94% after 10 days under UV exposure.

The MB01 lipopeptides showed good resistance to mediate strong acid of pH 1–5, and the lipopeptides activity was almost not affected (**Figure 8**). The lipopeptides activity reduced drastically to 0 when pH was less than 1. The residual rate still stayed above 50% when pH reached 13. However, it reduced to around 20% at pH 14. Considering the possible preservation conditions of industrialized production, the lipopeptides had good resistance to the pH change. The activities of lipopeptides retained above 94% after 10 days under UV exposure (**Figure 9**), which proved the lipopeptides also had good resistance to the UV.

## Conclusion

A bacterial strain MB01 producing cyclo-lipopeptide antibiotics was isolated from the Bohai Sea sediments, which had been identified as *Bacillus licheniformis* by the morphological, physiological, and biochemical identification and 16s rDNA sequence analysis. The purified lipopeptides achieved more than 3 cm of bacteriostatic zone in diameter, which proved that the lipopeptides from MB01 have a very strong capability against *Vibrio parahaemolyticus*, etc. The surfactins produced by MB01 were the surface active agents which had lipophilic group and hydrophilic group. Preliminary experiment on discharge of oil ring displayed that MB01 had the effect of degradation of oil (data not shown), and further research can be done on petroleum hydrocarbon degradation.

IR, amino acid analysis, LC-MS, FAB-MS, ESI-MS, PSD-MALDI-TOF-MS, and NMR were applied to understand the structure of the purified lipopeptides. Mass spectrometry is a new technology developed in recent years and has the advantages of high sensitivity, high accuracy, and low sample consumption. Purification methods in this study are mature with high efficiency. Acid sinking, extraction, ultrafiltration separation for crude separation, gel column and reverse phase C-18 column for fine separation, along with LC-ESI-MS/MS for measurement structure have been proved to be efficient methods to authenticate homologs (Kim Y. et al., 2004; Lee et al., 2008). Ultrafiltration and gel filtration can remove most of the impurities before the reversed-phase C18 purification. So a series of purified safactin homologs were detected after LC purification.

The lipopeptides from MB01 have very good stability, which could be related to the chemical ring structure. The lipopeptides have shown good resistance to pH, high temperature and UV and the bacteriostatic activity can be maintained for at least 24 h. The lipopeptides have the great advantage compared with the antimicrobial peptides (AMPs). Antibacterial peptides are considered to be effective, thermally stable and broad-spectrum fungicide, generally composed of 30–60 amino acids. However, AMPs will lose most of the antimicrobial activity under the condition of 80°C for 15 min. AMPs are fragile to pH change and can be disabled under pH < 4 or pH > 9 for 24 h (Boulanger et al., 2006; Wu et al., 2009; Kim et al., 2011; Silva et al., 2013). As a contrast, our lipopeptides can guarantee 100% of residual rate at 4°C for 10 days, and above 80% at 80°C for 10 days under the same conditions (**Figure 7**). This research reveals this MB01 lipopeptides have better pH stability (**Figure 8**). Protein denaturation will occur if AMPs are under UV irradiation (Brogden, 2005), however, UV resistance of the MB01 lipopeptides is very strong as a contrast (**Figure 9**).

This research can provide very useful references for industrialized application of the lipopeptides. Further research of the action mechanism of surfactins should be done to discover the effects of the lipopeptides on the cell membrane lipids of the different bacteria. Proteomic technology also can be applied to research the impact on the cell membrane proteins of the different bacteria.

## Author Contributions

YC performed all the experiments, coordinated the data analysis, and prepared the manuscript. XH and SL contributed in the experimental proposal and manuscript polishing. HM contributed in the experiment skills education. YM provided part of the research work suggestion and supervised the whole study. ML prepared part of the research materials.

## Conflict of Interest Statement

The authors declare that the research was conducted in the absence of any commercial or financial relationships that could be construed as a potential conflict of interest.
